# ADAR1 Facilitates HIV-1 Replication in Primary CD4^+^ T Cells

**DOI:** 10.1371/journal.pone.0143613

**Published:** 2015-12-02

**Authors:** Eloy Cuadrado, Thijs Booiman, John L. van Hamme, Machiel H. Jansen, Karel A. van Dort, Adeline Vanderver, Gillian I. Rice, Yanick J. Crow, Neeltje A. Kootstra, Taco W. Kuijpers

**Affiliations:** 1 Department of Experimental Immunology, Academic Medical Center (AMC), University of Amsterdam (UvA), Amsterdam, The Netherlands; 2 Sanquin Research, Landsteiner Laboratory and Center for Infection and Immunity (CINIMA) at the Academic Medical Center of the University of Amsterdam, Amsterdam, The Netherlands; 3 Center for Genetic Medicine Research, Children’s National Medical Center, Washington DC, United States of America; 4 Manchester Centre for Genomic Medicine, University of Manchester, Manchester Academic Health Sciences Centre (MAHSC), Manchester, United Kingdom; 5 INSERM UMR 1163, Laboratory of Neurogenetics and Neuroinflammation, Institut Imagine, Hôpital Necker, Paris, France; 6 Emma Children’s Hospital, Dept of Pediatric Hematology, Immunology and Infectious disease, AMC, UvA, Amsterdam, The Netherlands; Jackson Laboratory, UNITED STATES

## Abstract

Unlike resting CD4^+^ T cells, activated CD4^+^T cells are highly susceptible to infection of human immunodeficiency virus 1 (HIV-1). HIV-1 infects T cells and macrophages without activating the nucleic acid sensors and the anti-viral type I interferon response. Adenosine deaminase acting on RNA 1 (ADAR1) is an RNA editing enzyme that displays antiviral activity against several RNA viruses. Mutations in ADAR1 cause the autoimmune disorder Aicardi-Goutieères syndrome (AGS). This disease is characterized by an inappropriate activation of the interferon-stimulated gene response. Here we show that HIV-1 replication, in ADAR1-deficient CD4^+^T lymphocytes from AGS patients, is blocked at the level of protein translation. Furthermore, viral protein synthesis block is accompanied by an activation of interferon-stimulated genes. RNA silencing of ADAR1 in Jurkat cells also inhibited HIV-1 protein synthesis. Our data support that HIV-1 requires ADAR1 for efficient replication in human CD4^+^T cells.

## Introduction

Recent studies have begun to elucidate the role of host proteins in the innate immune response to infection with HIV-1. Of particular note, HIV-1 exploits the three prime repair exonuclease 1 (TREX1) protein to avoid the induction of type I interferons in CD4+ T cells and macrophages [1], whilst the deoxynucleoside triphosphohydrolase (SAMHD1) has been identified as a restriction factor limiting HIV-1 infection of myeloid lineage cells [2–4]. The genes encoding TREX1 [5] and SAMHD1 [6] are mutated in the human type I interferonopathy Aicardi-Goutieères syndrome (AGS). AGS is a rare genetic autoimmune disorder that can mimic congenital viral infection [5] and is characterized by an excessive production of interferon alpha (IFN-α) [7]. AGS can also be caused by dysfunction of the RNase-H2 endonuclease complex [8], the cytosolic double-stranded RNA (dsRNA) sensor MDA5 [9,10] and the dsRNA-editing enzyme ADAR1 [11]. All these proteins are important in nucleic acid metabolism and mutations in one of the AGS genes, i.e. TREX1, have been shown to lead to the accumulation of nucleic acids in cells, mimicking a viral infection and to the activation of the innate immune response [12–14].

ADAR1 activity has been studied in different types of viral infections, and both pro-viral [15,16] and anti-viral [17–19] activities have been reported and have been thoroughly reviewed elsewhere [20]. A highly selective editing of viral RNA mediated by ADAR1 has been demonstrated for hepatitis C virus (HCV) [19]. For HIV-1, the existing data are scarce and sometimes conflicting. ADAR1 has been reported to facilitate HIV-1 replication [21–23], whereas another study suggested that ADAR1 inhibits the production of viral proteins [24]. None of these investigations were carried out in human CD4+ T cells and thus little is known about the exact involvement of ADAR1 in the life cycle of HIV-1 in human CD4+ T cells.

## Methods

### Patient recruitment and cell isolation

AGS patients were recruited at Children’s National Medical Center, Washington DC, USA; Manchester Academic Health Science Centre, Manchester, UK; and the Academic Medical Center, Amsterdam, The Netherlands. ADAR1 mutation status and patient demographics have been described previously [11] and the information is summarized in Table 1. This study was conducted in accordance with the ethical principles set out in the declaration of Helsinki, written informed consent was obtained from each patient, and the Medical Ethics Committee of the Academic Medical Center and the Ethics Advisory Body of the Sanquin Blood Supply Foundation in Amsterdam (MEC07/234) approved the study.

**Table 1:** Demographic data, ancestry, and sequence alterations in *ADAR1* mutation–positive donors.

Patient	Age	Sex	Ancestry	Consanguinity	Nucleotide alteration	Exon	Amino-acid alteration
Patient 1	14	F	Norwegian	Non-consanguineous	c.[577C>G]+[2675G>A]	2, 9	p.[Pro193Ala]+[Arg892His]
Patient 2	4	M	European American	Non-consanguineous	c.3019G>A (het, *de novo*)	11	p.Gly1007Arg
Patient 3	9	F	Pakistani	Consanguineous	c.3337G>C (hom)	14	p.Asp1113His

M, male; F, female; het, heterozygous; hom, homozygous.

### Cell culture

PBMC were obtained from buffy coats from healthy blood donors. PBMC were isolated by Ficoll-Isopaque density gradient centrifugation and cultured in Iscove's modified Dulbecco medium (IMDM) supplemented with 10% (v/v) heat-inactivated fetal calf serum (FCS), penicillin (100 U/ml), streptomycin (100 μg/ml) and IL-2 (100 IU/ml) and maintained in a humidified 10% CO2 incubator at 37°C.

We used Jurkat T cells expressing CCR5 generated by retroviral transduction as previously published [25,26]. Cells were cultured in Roswell Park Memorial Institute Medium (RPMI) (Lonza, Basel, Switzerland) supplemented with 10% (v/v) heat-inactivated fetal calf serum (FCS), penicillin (100 U/ml) and streptomycin (100 μg/ml), in a humidified 5% CO2 incubator at 37°C. When indicated, cells were treated with a cocktail of RTIs including 10 μM AZT, 20 μM DDI, and 10 μM 3TC.

### Viruses

HIV-1YU2, HIV-1YU2-GFP, and VSV-G pseudotyped NL4-3.Luc.R-E-luciferase reporter viruses were produced by transfection of respectively pYU2, pYU2-GFP, or pNL4-3.Luc.R-E- in combination with pCMV-VSV-G in HEK293T cells. Lentiviral vectors (LV) were produced by co-transfection of pCMV-VSV-G, pMDLgp and pRSV-Rev and pLKO.1 constructs expressing ADAR1 shRNA in HEK293T cells. The following plasmids targeting ADAR1 were obtained from the The RNAi Consortium (TRC) library (Sigma, St Louis, MO, USA): TRCN0000050788 (clone d10), TRCN0000050789 (clone d11), TRCN0000050790 (clone d12), TRCN0000050791 (clone e1), TRCN0000050792 (clone e2). Vectors containing a non-targeting (NT) sequence of the human genome (NT-shRNA, SHC002, MISSION® Non-Target shRNA Control Vector) or green fluorescent protein (SHC003, MISSION® TurboGFP Control Vector) from Sigma were used as controls. Transfections were performed with the calcium phosphate method as described previously [27]. In short, plasmid DNA was diluted in 0.042M HEPES containing 0.15M CaCl2, subsequently mixed with an equal volume of 2× HEPES buffered saline pH 7.2, incubated at room temperature for 15 min and added to the culture medium. After 24h incubation in a humidified 3% CO2 incubator at 37°C, the culture medium was replaced and cultures were continued at 10% CO2 at 37°C. Virus was harvested at 48 and 72h after transfection and passed through a 0.22 μm filter. HIV-1 virus titers were quantified by determining the TCID50 on TZMBL or HEK293T. Lentiviral vector supernatant was concentrated by centrifugation at 50,000xg for 2.5h directly after harvesting. Equal amounts of lentiviral vector as determined by p24 ELISA were used to inoculate target cells.

### Lentiviral transduction and HIV-1 infection

To obtain Jurkat T cells with a stable ADAR1 knockdown, the cells were inoculated with either the LV-shRNA NT-control or the LV-shRNA targeting ADAR1 at a MOI of 10. Cells were subsequently cultured in the presence of puromycin (2 μg/ml) for several passages to obtain a stable transduced cell line. Jurkat T cell lines and IL-2 stimulated PBMCs were infected with YU2, YU2-GFP or the VSV-G pseudotyped NL4-3.Luc.R-E- luciferase reporter viruses which were treated with DNAse (200 ng/ml) (Promega, Madison WI, USA) for 45 min at 37°C in medium containing 6 mM MgCl2 at a MOI of 0.1 and 1. Three hours post-infection the cells were washed with medium and the medium was replaced. *pol* proviral DNA copies and *pol*, *Gag* and *GFP* mRNA levels were analysed 48h post infection. Gag p24 levels in the culture supernatant were determined respectively 7 days post infection by an in-house enzyme-linked immunosorbent assay [28]. Luciferase activity was determined 48h post infection as a measure for HIV-1 replication, by adding 25 μl substrate (0.83 mM ATP, 0.83 mM D-luciferin (Duchefa, Haarlem, The Netherlands), 18.7 mM MgCl2, 0.78 μM Na2H2P2O7, 38.9 mM Tris pH 7.8, 0.39% (v/v) glycerol, 0.03% (v/v) Triton X-100, and 2.6 μM dithiothreitol) directly to the culture medium. Luminescence was measured for 1 s per well using a luminometer (Berthold, Bad Wildbad, Germany).

### Nucleic acid isolation and quantitative PCR

Total DNA and RNA were isolated using the Allprep DNA/RNA mini kit (QIAGEN). Reverse transcription of mRNA was performed using the Roche Transcriptor First Strand cDNA Synthesis kit (Roche) using a maximum of 1 μg total RNA and random hexamer primers. The primers used for qPCR and real time qPCR were ADAR1 p110 fw 5’-GGCAGCCTCCGGGTG-3, rv 5’-CTGTCTGTGCTCATAGCCTTGA-3’; ADAR1 p150 fw 5’-CGGGCAATGCCTCGC-3, rv 5’- AATGGATGGGTGTAGTATCCGC-3’; GAPDH fw 5’-TGCACCACCAACTGCTTAGC-3’, rv 5’- GGCATGGACTGTGGTCATGA-3’; ACTB fw 5’-GCTCCTCCTGAGCGCAAG-3’, rv 5’-CATCTGCTGGAAGGTGGACA-3’; OAS1 fw 5’-CTGACTCCTGGCCTTCTATG-3’, rv 5’- GGCTGTGGAGAATGTTATCTATG-3’; IFI27 fw 5’-TAGCAGCCAAGATGATGTCCG-3’, rv 5’- AGAGTCCAGTTGCTCCCAGTGA-3’; IFI44L fw 5’-ATGTTCAGCTGTACCCTCCCAC-3’, rv 5’- TGCCTACACTGCACTTCCTGTC-3’; ISG15 fw 5’-CATCTTTGCCAGTACAGGAGCT-3’, rv 5’- ACACCTGGAATTCGTTGCC-3’; RSAD2 fw 5’-AAGTCCATCCTGGATGTTGGTG-3’, rv 5’- TTCCGCTCTACCAATCCAGCT-3’; eGFP fw 5’- GATCGATCGAATTCATGGTGAGCAAGGGCGAGGA-3’, rv 5’- GATCGATCCTCGAGTTACTTGTACAGCTCGTCCATGCCG-3’; Gag fw 5’- CAGCAATCAGGTCAGCCAAAATTAC-3’, rv 5’-CTTCTACTACTTTTACCCATGC-3’; Pol fw 5’- CTTCTAAATGTGTACAATCTAGTTGCC-3’, rv 5’-TGATTTTAACCTGCCACCTGTAGTAG-3’; probe: (FAM)-CTGTGATAAATGTCAGCTAAAAGGAGAAGCCA-(TAMRA). qPCRs were performed on a Lightcycler 480 II (Roche) using SYBR Green I Master or Probes Master (Roche) and gene expression values were obtained using Roche’s LightCycler® relative quantification software (release 1.5.0).

### Flow cytometry

Expression of surface-bound receptors and viral GFP was detected by flow cytometry, with the commercially available monoclonal antibodies 7-AAD, CD3 Pacific Blue, and CD4 PE-Cy7 or V500 (BD Biosciences, San Jose, CA). Samples were analyzed on a FACSCanto II flow cytometer equipped with FACSDiva software (BD Biosciences, San Jose, CA, USA). Cells were gated based on their forward and side scatter, and 20,000 gated events were collected per sample. Data were analyzed using FlowJo Version 7.6.4 software (TreeStar).

### Western blot

Cells were lysed in RIPA-buffer (150 mM NaCl, 1% Triton X-100, 0.5% sodium deoxycholate, 0.1% SDS, 50 mM Tris, pH 8.0) containing Complete® EDTA free protease inhibitor (Roche). After adding NuPAGE LDS 4x sample buffer (Invitrogen) and 0.1M DTT, samples were heated at 95°C for 10 min. The Odyssey Protein Weight Marker was used as a size reference (LI-COR, Lincoln, NE, USA). Proteins were separated by SDS-PAGE (NuPAGE 10% Bis-Tris precast gel and MES SDS running buffer (Invitrogen) and transferred to a nitrocellulose membrane (Protran, Schleicher & Schuell, Dassel, Germany) using NuPAGE transfer buffer. After blocking for 2h with PBS containing 5% Protifar (Nutricia, Schiphol, The Netherlands) and 0.5% bovine serum albumin, the blot was incubated with anti ADAR1 antibody to detect ADAR1 (1:1000; Sigma-Aldrich St. Louis, MO, USA), eIF2α (1:1000; mAb #2103, Cell Signalling, Beverly, MA, USA), Phospho-eIF2α (Ser51) (1:1000; mAb #3597, Cell Signalling, Beverly, MA, USA), and SC-1616 anti-β-actin antibody (1:200; Santa Cruz Biotechnology, Santa Cruz, CA, USA) to detect β-actin. IRDye 680 conjugated Donkey anti-Rabbit IgG (1:15000; 926–32223, LI-COR, Lincoln, NE, USA) and IRDye 680LT conjugated Donkey anti-Goat IgG (1:15000; 926–32224, LI-COR) were used as secondary antibodies to visualize expression using the Odyssey infrared image system (LI-COR). Image J software was used to quantify protein expression.

### Statistics

Data were analyzed using GraphPad Prism 6.0 (GraphPad Software Inc., San Diego, CA, USA). All experiments were performed at least 3 independent times, unless otherwise specified. The variable distribution was assessed by the Kolmogorov-Smirnov test. When test distribution was normal a Student t-test was used to determine differences between 2 groups. When distribution was not normal, a non-parametric Mann-Whitney test was used to determine intergroup differences. A P-value of < 0.05 was considered statistically significant at a 95% confidence level.

## Results and Discussion

**Fig 1:**
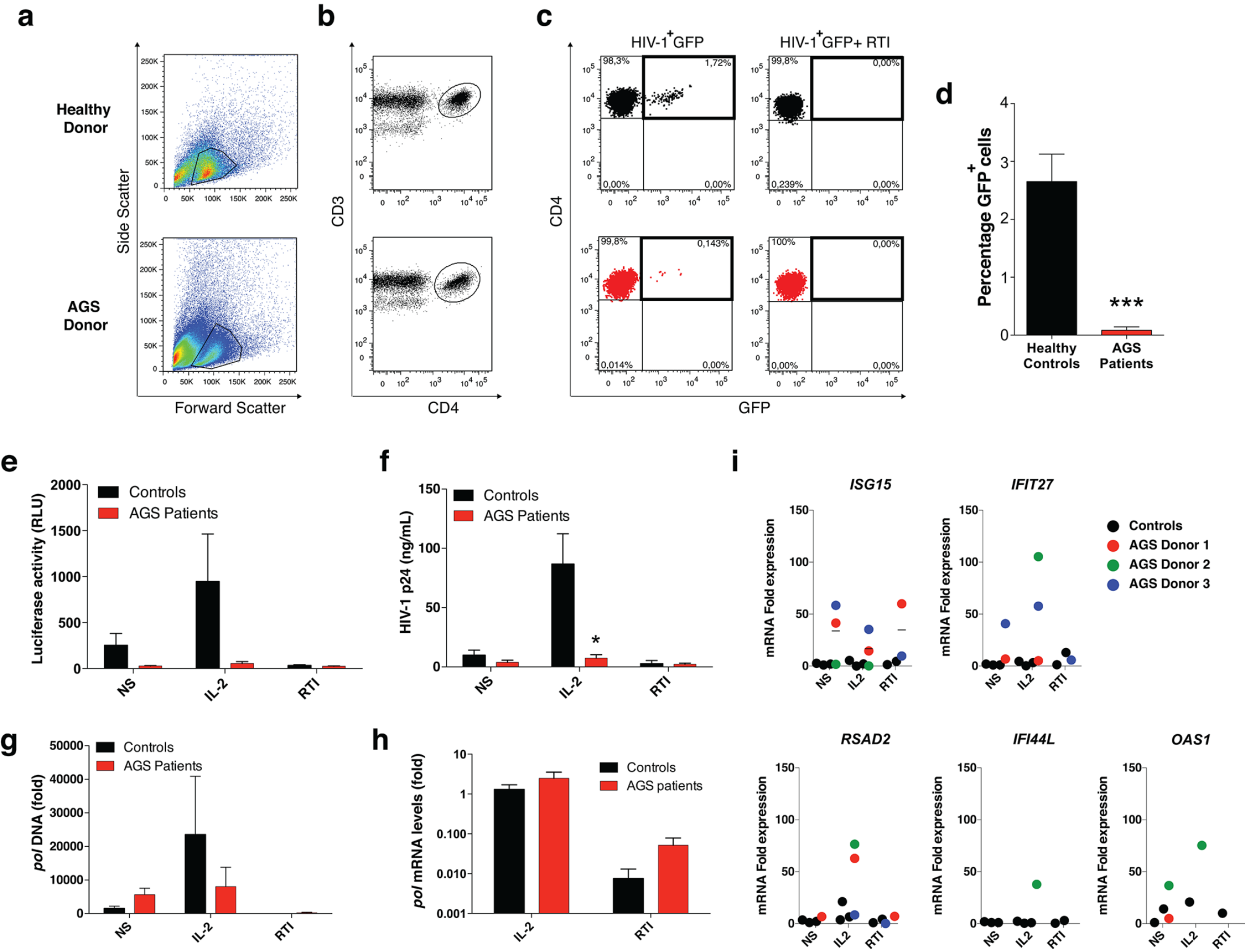
ADAR1 deficiency restricts HIV-1 replication in activated human CD4+ T cells. (a-b) PBMCs from healthy donors (n = 4) and ADAR1-mutated patients (n = 3) were challenged with equivalent infectious units of HIV-1 GFP virus and analyzed on day 5 after infection. The reverse transcriptase inhibitor (RTI) cocktail (10μM AZT, 20μM DDI, and 10μM 3TC) was used as specificity control. (a) Representative dot plots of flow cytometry analysis from PBMCS of an AGS patient and a healthy control. The viable cells (gate, left graphs) were stained and selected for CD3+CD4+ expression (b). The percentages of infected (GFP+) CD4+ T cells (c) are shown in the upper right quadrant. (d) Bar graph representing the mean±s.e.m. of GFP+ infected cells (n = 3), ***P<0.001. (e) Bars represent luciferase activity in relative luciferase units (RLU) of cells infected with a VSV-G virus. The activity was measured in non-stimulated (NS), IL-2-stimulated or RTI-treated cells. Bars represent mean±s.e.m (n = 3). (f) Bar graphs represent mean±s.d (n = 3) of HIV-1 p24 protein amounts released into the cell culture supernatant on day 5 after infection, *P<0.05. Relative quantification of viral DNA pol (g) and viral pol mRNA (h) expressed as fold versus NS control cells. (i) Relative quantification of ISG15, IFIT27, RSAD2, IFIT44L, and OAS1 from HIV-1-infected PBMCs from AGS patients (n = 3) and healthy donors (n = 3) presented as fold over NS control expression.

Here we have studied the role of ADAR1 in HIV-1 replication using primary T cells obtained from AGS patients carrying known ADAR1 mutations [11], and thus lacking fully functional ADAR1 activity. Infection of CD3+CD4+ T cells with the HIV-1 GFP reporter virus was analyzed by flow cytometry (Fig 1A–1C). CD4+ T cells from healthy donors supported productive infection with HIV-1 GFP, yielding >2.5% of GFP positive CD3+CD4+ T cells 5 days post-infection (Fig 1D). In contrast, CD4+ T cells from AGS patients were refractory to infection with HIV-1 GFP. As expected, viral infection of CD4+ T cell cultures was inhibited in the presence of reverse transcriptase inhibitors (RTI) (Fig 1C). Infection of peripheral blood mononuclear cells (PBMCs) from AGS patients with a VSV-G pseudotyped luciferase reporter virus was also absent when compared to the healthy controls (Fig 1E), indicating that HIV-1 replication in PBMC from AGS patients is not restricted at the level of viral entry.

We determined which step of the viral replication cycle was impaired in cells from AGS patients. In agreement with the results described above, efficient viral replication in PBMC from healthy donors could be observed when virus production in the culture supernatant was analyzed using a p24 ELISA. However, p24 production in the culture supernatant from AGS PBMCs remained below the detection limit (Fig 1F). Next, we analyzed whether the process of HIV-1 reverse transcription and proviral transcription could occur efficiently in PBMC from ADAR1-deficient patients. We observed no differences in pol proviral DNA (Fig 1G) and pol mRNA levels (Fig 1H) in PBMC from AGS patients as compared to healthy controls. These data indicate that HIV-1 replication is impaired at a post-transcriptional step.

AGS is characterized by high levels of IFN-alpha, which usually invokes the activation of interferon-stimulated genes (ISGs) [29]. ISGs are found upregulated in PBMCs from patients carrying mutations in ADAR1 [11]. We also observed an upregulation in mRNA expression of *ISG15*, *IFIT27*, *RSAD2*, and *OAS1* in AGS patient cells infected with HIV-1 compared to cells from healthy donors under similar conditions (Fig 1I).

**Fig 2:**
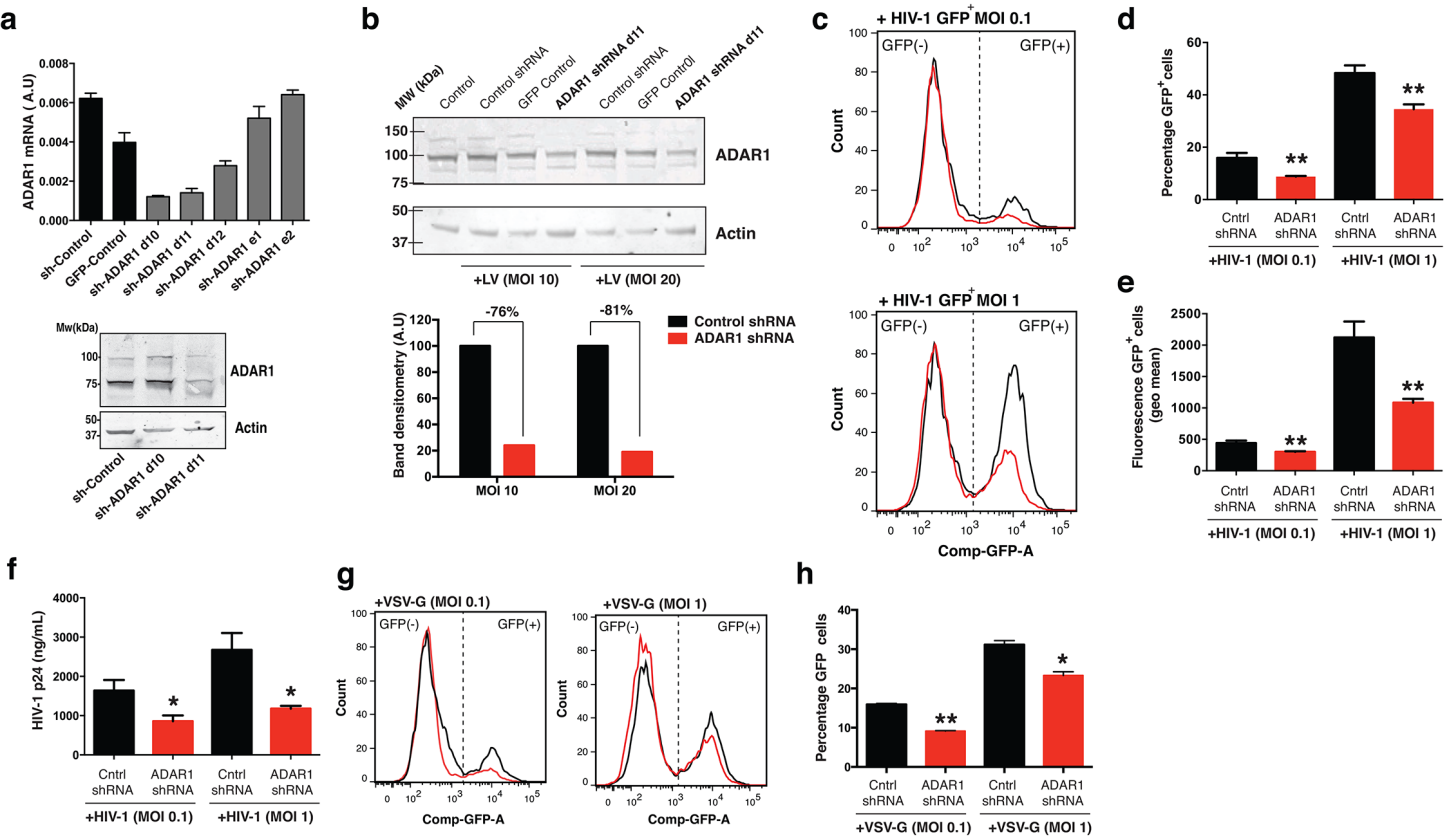
Silencing ADAR1 restricts HIV-1 replication in Jurkat T cells. (a) Jurkat T cells were stable transduced with shRNA control, GFP control or ADAR1 shRNAs. Bar graph shows normalized ADAR1 mRNA levels. Panel shows western blot of the two best shRNA targeting ADAR1. (b) Cells were transduced at MOI 10 or 20. Panels show representative western blot of ADAR1 protein reduction in ADAR1 shRNA transduced cells. Bar graphs show densitometry analysis of bands compared to Control shRNA. Actin was used as loading control. (c) Control shRNA (n = 4) and ADAR1 shRNA (n = 4) transduced Jurkat T cells were challenged with equivalent infectious units of HIV-1 GFP virus (MOI 0.1 or 1) and analyzed by FACS on day 5 after infection. Panels show representative dot plots of FACS analysis from transduced cells. The viable cells (gate, left graphs) were analyzed for GFP fluorescence. Histograms show GFP signal of Control shRNA (black line) and ADAR1 shRNA cells (red line) after HIV1-GFP infection at different MOIs. (d) Bar graph representing the mean±s.d. of GFP+ infected cells (n = 4), **P<0.01. (e) Bar graph representing the mean±s.d. of GFP fluorescence of infected cells (n = 4), **P<0.01. (f) Bar graphs represent mean±s.d. (n = 3) of HIV-1 p24 protein amounts released into the cell culture supernatant on day 5 after infection, *P<0.05. (g) Histograms show GFP signal of control shRNA (black line) and ADAR1 shRNA cells (red line) after VSV-G-GFP infection at different MOIs. (h) Bar graph representing the mean±s.d. of GFP+ infected cells (n = 3) **P<0.01, *P<0.05.

To confirm that ADAR1 supports HIV-1 replication in T lymphocytes, we analyzed the effect of ADAR1 knockdown on HIV-1 replication in CCR5-expressing Jurkat T cells. Normally Jurkat cells do not express CCR5; for that reason we used a stable CCR5-expressing Jurkat cell line [30]. First, HEK293T cells were transduced with lentiviral vectors expressing a non-targeting (NT) control shRNA or shRNAs targeting ADAR1 gene transcription (Fig 2A and S1 Fig). Next, the most efficient ADAR1 shRNA clone (clone d11) was subsequently used on Jurkat T cells and resulted in a stable reduction of ADAR1 expression of 81% (Fig 2B and S1 Fig). These cells were infected with a HIV-1 GFP reporter virus at two different multiplicities of infection (MOIs), and viral replication was analyzed 5 days post-infection by flow cytometry (Fig 2C). We observed a decrease in the number of GFP+ cells and fluorescence intensity in the ADAR1 knockdown cells compared to NT control cells (Fig 2D and 2E). When these experiments were repeated with a replication-competent HIV-1 variant, we also observed a decrease in viral replication as measured by p24 release into the culture supernatant of these ADAR1 knockdown cells (Fig 2F). Similar results were obtained using a VSV-G pseudotyped virus (Fig 2G and 2H).

**Fig 3:**
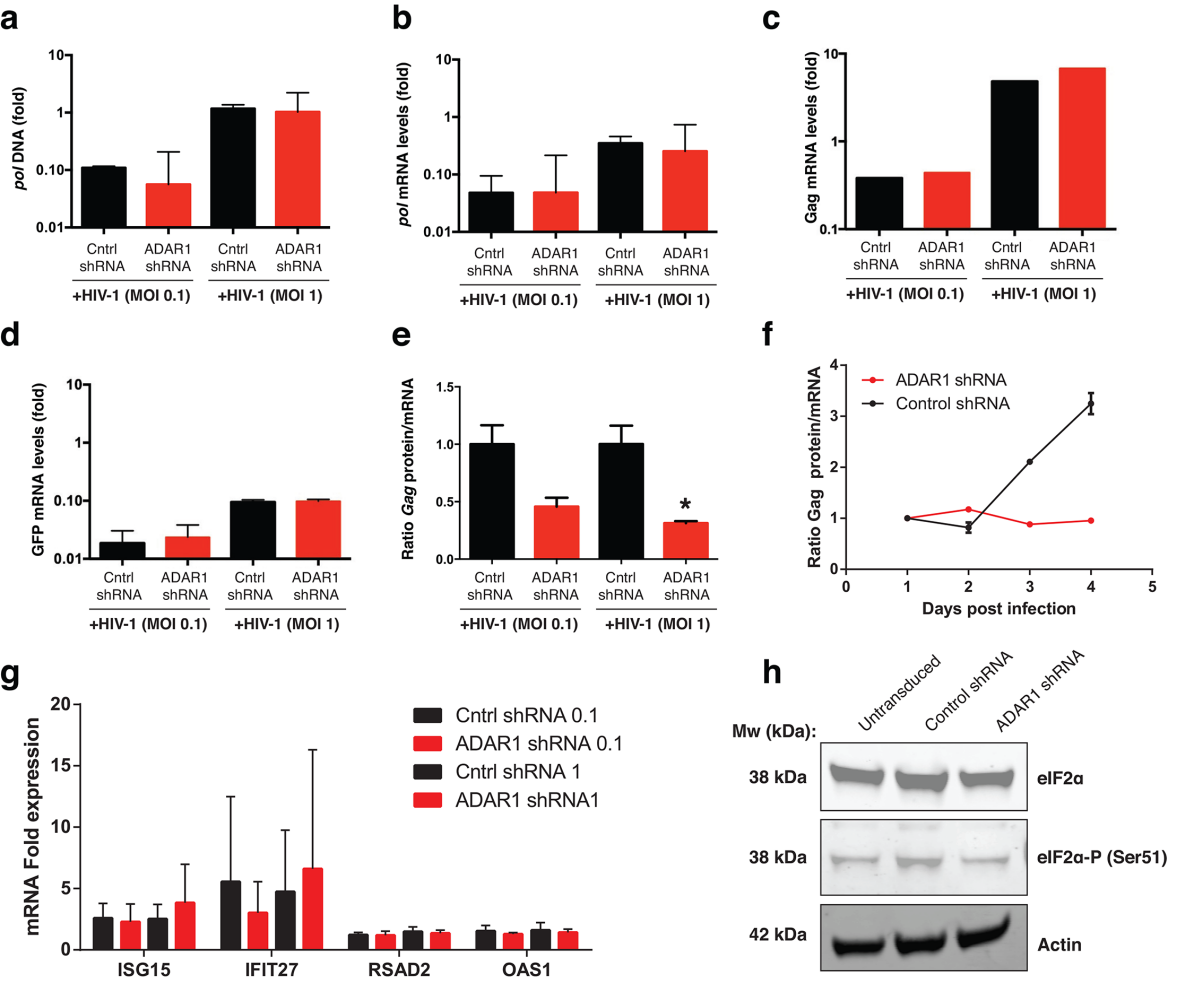
Relative quantification (mean±s.e.m) of viral DNA pol (a) and viral pol mRNA (b) expressed as fold versus NT-shRNA control cells (n = 3). (c) Gag (n = 2) and (d) GFP (n = 3) mRNA expressed as fold versus NT-shRNA control cells (e) Bar graphs show the ratio of Gag protein versus Gag mRNA levels on infected cells (n = 3), *P<0.05. (f) Time course ratio expression of Gag protein versus Gap mRNA from day 1 to day 5 after infection (n = 2 =). (g) Relative quantification of ISG15, IFIT27, RSAD2, IFIT44L, and OAS1 of control shRNA and ADAR1 shRNA cells. (h) Western blot of eIF2α and the phosphorylated form of eIF2α. Actin was used as loading control.

We then investigated which step of the viral replication cycle is facilitated by ADAR1. Proviral DNA and viral mRNA expression was determined by qPCR 48h post infection (MOI 0.1 and 1). We observed no difference in pol proviral DNA levels and pol mRNA expression in the ADAR1 knockdown cells when compared to the NT control cells (Fig 3A and 3B). In addition, no differences were observed in Gag and GFP mRNA expression levels (Fig 3C and 3D). These results indicate that HIV-1 replication occurs efficiently in ADAR1 knockdown cells up to the point of viral transcription. When the level of Gag mRNA expression was correlated to the level of Gag protein expression, a clear decrease in protein production per viral gag transcript was observed in ADAR1 knockdown cells (Fig 3E) and this difference was already evident after 3 days post-infection (Fig 3F). Next, we analyzed ISG mRNA expression in CCR5-expressing Jurkat cells upon ADAR1 knockdown and observed no changes in *ISG15*, *IFIT27*, *RSAD2*, and *OAS1* expression levels (Fig 3G). These data, together with the data from the primary cells obtained from the AGS patients, suggest that these ISGs are not responsible for the block in HIV-1 replication observed in ADAR1 deficient cells.

The actual mechanism by which ADAR1 interferes with HIV-1 replication is not fully understood. ADAR1 prevents phosphorylation of RNA-activated protein kinase (PKR) and thus phosphorylation of eukaryotic translation initiation factor 2 (eIF2α), which is important for the initiation of mRNA translation [23]. Here we show that mutations in ADAR1 or ADAR1-knockdown cells restrict HIV-1 replication at the level of protein translation, possibly through the inhibition of PKR and eIF2α phosphorylation. However, in our CCR5-expressing Jurkat T cells, the inhibition of HIV-1 replication could not be related to increasing levels of phosphorylated eIF2α upon ADAR1 knockdown as measured by western blot 2 days after infection (Fig 3H and S2 Fig). Nonetheless, the lack of increased phosphorylation of eIF2α at such early time point does not exclude PKR inhibition as the main key player in ADAR1-mediated blocking of HIV-1 virus replication. In fact, it has recently been shown that the PKR activator (PACT) contributes to PKR inhibition during HIV-1 replication and that the TAR RNA binding protein (TRBP) interacts with ADAR1 resulting in an increased replication of HIV [31].

Our results undoubtedly show a clear decrease in Gag protein with no decrease in Gag mRNA. This may indicate a post-transcriptional regulation of Gag, which can occur through RNA editing or decreased translation. This latter phenomenon is compatible with the inhibition of PKR activation that has been described for HIV [23,31] but also for other viruses such as VSV [32], Measles virus [33,34] and human T-cell lymphotropic virus (HTLV-I) [35]. ADAR1 activity through RNA editing has also been described for HIV [21,22] but also for other viruses such as equine infectious anemia virus (EIAV) [15].

In summary, our results demonstrate that ADAR1 facilitates HIV-1 replication in human primary CD4+ T cells by supporting efficient viral protein synthesis, clarifying the discrepancy of previous studies [20–24,31]. Unlike TREX1 exonuclease, which attenuates the innate immune response to HIV-1 by clearing viral DNA transcripts [1], ADAR1 seems to facilitate HIV-1 replication by permitting efficient viral protein synthesis. Our study demonstrates a role for ADAR1 in HIV-1 replication in primary CD4+ T cells and may contribute to the development of therapeutics against HIV/AIDS by targeting a novel cellular pathway important for HIV-1 replication.

## Supporting Information

S1 Fig Original ADAR1 Western Blots. (TIFF)

S2 Fig Original eIF2alpha Western Blots. (TIFF)
