# Supplementary figures and images for: ADAR1 Facilitates HIV-1 Replication in Primary CD4+ T Cells
Source: PLoS One. 2015 Dec 2;10(12):e0143613. doi: 10.1371/journal.pone.0143613 (PMC4667845; doi:10.1371/journal.pone.0143613)

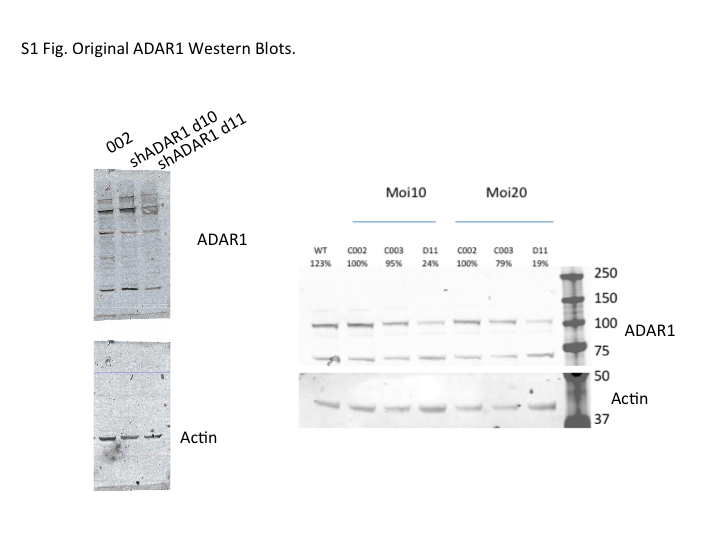

Supplement: S1 Fig — (TIFF) [file pone.0143613.s001.tiff]

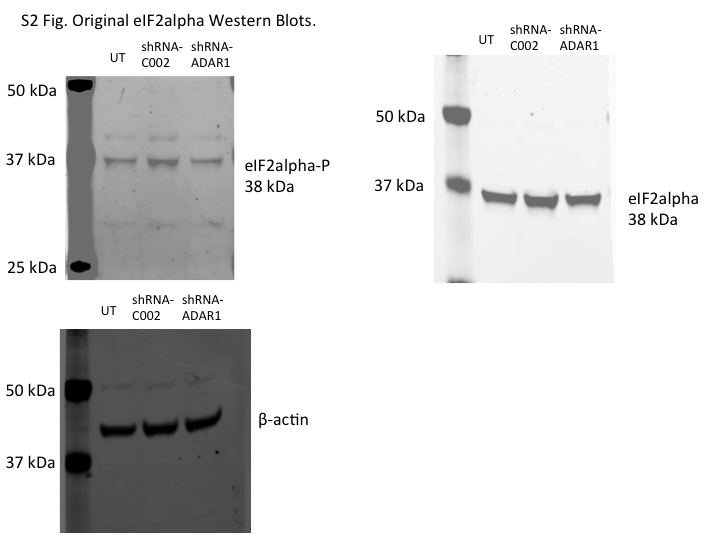

Supplement: S2 Fig — (TIFF) [file pone.0143613.s002.tiff]
